# Exposure to community violence and Callous-Unemotional traits in young children: the role of positive parenting

**DOI:** 10.1192/j.eurpsy.2022.584

**Published:** 2022-09-01

**Authors:** D. Obando, N. Wright, J. Hill

**Affiliations:** 1 Universidad de La Sabana, Psychology, Chía, Cundinamarca, Colombia; 2 Kings College, Biostatistics & Health Informatics, Institute Of Psychiatry, Psychology & Neuroscience, London, United Kingdom; 3 University of Reading, Psychology And Clinical Language Sciences, Reading, United Kingdom

**Keywords:** Low-middle-income country, Callous-unemotional traits, Exposure to community violence, Positive parenting

## Abstract

**Introduction:**

Studies regarding environmental contributions on callous-unemotional (CU) traits in children have informed about the protective role of positive parenting. However, it has not been explored whether findings from these studies -mostly conducted in High-Income Countries- can be generalised to Low-Middle-Income Countries (LMICs). Exposure to community violence is common in LMICs and is associated with emotional and behavioural problems in children. Therefore, it may represent an environmental risk factor for CU traits.

**Objectives:**

This prospective study explores whether positive parenting has a protective role in relation to CU traits in young Colombian children whose families have been exposed to community violence.

**Methods:**

We assessed 235 families with children at age 3.5 years, from three contrasting regions of Colombia, using observations of mother-child interactions and maternal reports of community violence at ages 3.5 and 5.0 years.

**Results:**

Hierarchical multiple linear regression models indicated that maternal positivity at 3.5 years was associated with lower CU traits at age 5.0 years only in children of families exposed to community violence (interaction term p= .001). In the exposed group maternal positivity explained 10% of the variance (β= -.34, p= .001) with low positivity associated with elevated CU traits and high positivity with low CU traits. Maternal praise was not associated with CU traits. However, maternal negativity during play was associated with elevated CU traits as a main effect.

**Conclusions:**

Based on these findings, whether or not exposure to community violence is associated with elevated CU traits depends on maternal positivity, with low positivity creating vulnerability, and high positivity, resilience.

**Disclosure:**

No significant relationships.

